# Supplementary material for: Aerosol-generating behaviours in speech pathology clinical practice: A systematic literature review
Source: PLoS One. 2021 Apr 28;16(4):e0250308. doi: 10.1371/journal.pone.0250308 (PMC8081183; doi:10.1371/journal.pone.0250308)
Supplement: S1 File — (PDF) [file pone.0250308.s002.pdf]

THE UNIVERSITY OF SYDNEY

# Aerosol-Generating Behaviours in Speech Pathology: A Systematic Literature Review

DLVP Dr Liang Voice Program

## Review Process

1. Records Collected From Initial Search = 8,288
2. Records Screened (following duplicate removal) = 6,093
3. Irrelevant Records Excluded = 5,933
4. Full Text Articles Screened (with irrelevant records excluded) = 160
5. Studies Included In Review = 39

24 RESEARCH STUDIES

12 REVIEW ARTICLES

3 CLINICAL GUIDELINES

39 RECORDS

## Types of Aerosol Generating Behaviours

Voice, Resonance and Motor Speech

### Level of Evidence (GRADE)

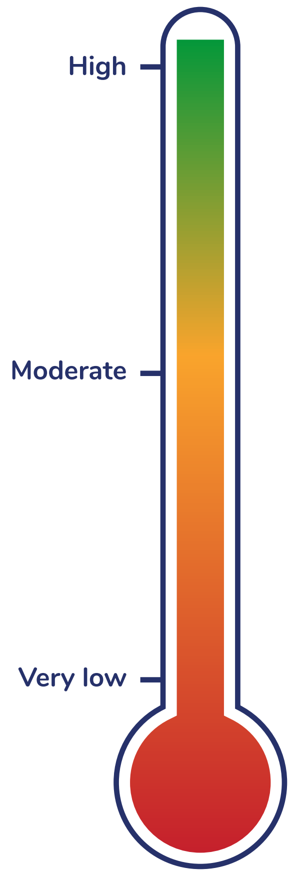


### Number of Records and GRADE Rating for each AGB

- Coughing= **31**
- Speaking= **21**
- Breathing= **16**
- Singing= **4**
- Sustained Phonation= **3**
- Loud Voicing= **3**

### Aerosol-Generating Behaviours

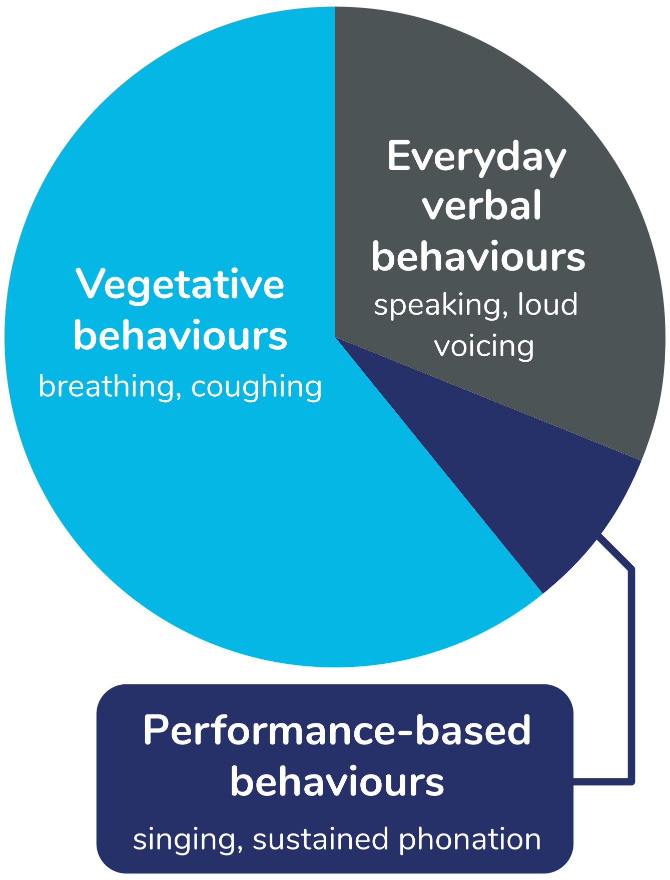


## Conclusions

**Six behaviours** were identified as **aerosol-generating**

Behaviours could be classed as **vegetative, everyday verbal** and **performance-based**

**Range** in certainty of **evidence** from **very low** to **moderate** with variation in research design and variables

- **Higher** levels of **evidence** were found for AGBs with a **larger number** of **studies**
- More high-level research is **urgently** needed across **all AGBs** to inform community practices.
