# Supplementary material for: Aerosol-generating behaviours in speech pathology clinical practice: A systematic literature review
Source: PLoS One. 2021 Apr 28;16(4):e0250308. doi: 10.1371/journal.pone.0250308 (PMC8081183; doi:10.1371/journal.pone.0250308)
Supplement: S2 File — (PDF) [file pone.0250308.s003.pdf]

S2 File. Search Strategy (Scopus, Medline, Embase, Web of Science, CINAHL and PubMed Central)

**Search Concepts**	**Terms associated with each concept**
Coronavirus	coronavirus OR corona AND virus OR sars-cov-2 OR 2019-ncov OR novel AND coronavirus 2019 OR covid-19 OR severe AND acute AND respiratory AND syndrome AND virus OR severe AND acute AND respiratory AND syndrome AND coronavirus 2 OR sars OR mers
Speech Pathology	speech AND language AND patholog* OR speech AND language AND patholog* OR speech AND patholog* OR speech AND therap* OR speech AND language AND therap* OR speech AND language AND therap* OR slp* OR slt* OR voice AND therap*
Speech Pathology Clinical Tasks	phonat* OR voice OR voc* OR breath* OR cough* OR speech* OR speak* OR talk* OR airway* OR articulat* OR labi* OR laryn* OR lingual* OR nasal* OR naso* OR oral* OR reading AND passage* OR rainbow AND passage OR grandfather AND passage OR vowel AND onset* OR phrase* OR cape-v OR consensus AND auditory AND perceptual AND evaluation AND of AND voice OR spasmodic AND dysphonia OR maximum AND phonat* AND time* OR vowel OR pitch* OR pitch AND range OR loud* OR soft* OR dynamic AND range OR count* OR sing* OR fricative* OR plosive* OR glottal AND stroke* OR resona* OR motor AND speech OR speech AND motor
Aerosols	aerosol* OR aerosol-generat* OR agp* OR expirat* OR droplet* OR partic* OR airborne* OR air-carr* OR air AND turbulence
Transmission Risk	hazard* OR risk* OR contaminat* OR expos* OR carr* OR transmi* OR microorganism* OR infect* OR viral AND load OR virus AND shed* OR bacteria* AND penetrat*
Risk Management	risk AND manag* OR control* OR risk AND reduc* OR restrict* OR avoid* OR prevent* OR protect* OR complian* OR adhere* OR recommend* OR disease AND prevention
Strategies for Risk Management	facemask* OR face AND mask* OR respirator* OR face AND shield OR personal AND protective AND equipment OR ppe OR distanc* OR eye AND protect* OR handwash* OR doff* OR clinical AND practice AND guideline*

Search No.	Concept areas	Terms used in search
1	Coronavirus and Speech Pathology	(coronavirus OR corona AND virus OR sars-cov-2 OR 2019-ncov OR novel AND coronavirus 2019 OR covid-19 OR severe AND acute AND respiratory AND syndrome AND virus OR severe AND acute AND respiratory AND syndrome AND coronavirus 2 OR sars OR mers) AND (speech AND language AND patholog* OR speech AND language AND patholog* OR speech AND patholog* OR speech AND therap* OR speech AND language AND therap* OR speech AND language AND therap* OR slp* OR slt* OR voice AND therap*)
2	Coronavirus and Speech Pathology Clinical Tasks	(coronavirus OR corona AND virus OR sars-cov-2 OR 2019-ncov OR novel AND coronavirus 2019 OR covid-19 OR severe AND acute AND respiratory AND syndrome AND virus OR severe AND acute AND respiratory AND syndrome AND coronavirus 2 OR sars OR mers) AND (phonat* OR voice OR voc* OR breath* OR cough* OR speech* OR speak* OR talk* OR airway* OR articulat* OR labi* OR laryn* OR lingual* OR nasal* OR naso* OR oral* OR reading AND passage* OR rainbow AND passage OR grandfather AND passage OR vowel AND onset* OR phrase* OR cape-v OR consensus AND auditory AND perceptual AND evaluation AND of AND voice OR spasmodic AND dysphonia OR maximum AND phonat* AND time* OR vowel OR pitch* OR pitch AND range OR loud* OR soft* OR dynamic AND range OR count* OR sing* OR fricative* OR plosive* OR glottal AND stroke* OR resona* OR motor AND speech OR speech AND motor)
3	Speech Pathology and Aerosols	(speech AND language AND patholog* OR speech AND language AND patholog* OR speech AND patholog* OR speech AND therap* OR speech AND language AND therap* OR speech AND language AND therap* OR slp* OR slt* OR voice AND therap*) AND (aerosol* OR aerosol-generat* OR agp* OR expirat* OR droplet* OR partic* OR airborne* OR air- carr* OR air AND turbulence)
4	Speech Pathology and Transmission Risk	(speech AND language AND patholog* OR speech AND language AND patholog* OR speech AND patholog* OR speech AND therap* OR speech AND language AND therap* OR speech AND language AND therap* OR slp* OR slt* OR voice AND therap*) AND (hazard* OR risk* OR contaminat* OR expos* OR carr* OR transmi* OR microorganism* OR infect* OR viral AND load OR virus AND shed* OR bacteria* AND penetrat*)
5	Speech Pathology and Risk Management	(speech AND language AND patholog* OR speech AND language AND patholog* OR speech AND patholog* OR speech AND therap* OR speech AND language AND therap* OR speech AND language AND therap* OR slp* OR slt* OR voice AND therap*) AND (risk AND manag* OR control* OR risk AND reduc* OR restrict* OR avoid* OR prevent* OR protect* OR complian* OR adhere* OR recommend* OR disease AND prevention)

6	Speech Pathology and Strategies for Risk Management	(speech AND language AND patholog* OR speech AND language AND patholog* OR speech AND patholog* OR speech AND therap* OR speech AND language AND therap* OR speech AND language AND therap* OR slp* OR slt* OR voice AND therap*) AND (facemask* OR face AND mask* OR respirator* OR face AND shield OR personal AND protective AND equipment OR ppe OR distanc* OR eye AND protect* OR handwash* OR doff* OR clinical AND practice AND guideline*)
7	Speech Pathology Clinical Tasks and Aerosols	(phonat* OR voice OR voc* OR breath* OR cough* OR speech* OR speak* OR talk* OR airway* OR articulat* OR labi* OR laryn* OR lingual* OR nasal* OR naso* OR oral* OR reading AND passage* OR rainbow AND passage OR grandfather AND passage OR vowel AND onset* OR phrase* OR cape-v OR consensus AND auditory AND perceptual AND evaluation AND of AND voice OR spasmodic AND dysphonia OR maximum AND phonat* AND time* OR vowel OR pitch* OR pitch AND range OR loud* OR soft* OR dynamic AND range OR count* OR sing* OR fricative* OR plosive* OR glottal AND stroke* OR resona* OR motor AND speech OR speech AND motor) AND (aerosol* OR aerosol-generat* OR agp* OR expirat* OR droplet* OR partic* OR airborne* OR air-carr* OR air AND turbulence)
8	Speech Pathology Clinical Tasks and Transmission Risk	(phonat* OR voice OR voc* OR breath* OR cough* OR speech* OR speak* OR talk* OR airway* OR articulat* OR labi* OR laryn* OR lingual* OR nasal* OR naso* OR oral* OR reading AND passage* OR rainbow AND passage OR grandfather AND passage OR vowel AND onset* OR phrase* OR cape-v OR consensus AND auditory AND perceptual AND evaluation AND of AND voice OR spasmodic AND dysphonia OR maximum AND phonat* AND time* OR vowel OR pitch* OR pitch AND range OR loud* OR soft* OR dynamic AND range OR count* OR sing* OR fricative* OR plosive* OR glottal AND stroke* OR resona* OR motor AND speech OR speech AND motor) AND (hazard* OR risk* OR contaminat* OR expos* OR carr* OR transmi* OR microorganism* OR infect* OR viral AND load OR virus AND shed* OR bacteria* AND penetrat*)

Limits applied to all searches:
Date: January 1940- current
Language: English Only
Animal Studies: Excluded
Children ≤ 12 years old: Excluded
