# Supplementary material for: Aerosol-generating behaviours in speech pathology clinical practice: A systematic literature review
Source: PLoS One. 2021 Apr 28;16(4):e0250308. doi: 10.1371/journal.pone.0250308 (PMC8081183; doi:10.1371/journal.pone.0250308)
Supplement: S3 File — (PDF) [file pone.0250308.s004.pdf]

S3 File. Data Extraction Tables *Summary Table 1:*

Extracted Data from Research Studies

Authors	Article title	Year	AGPs under investigation	Study type	Sample size	Outcome measures	Main findings
Duguid JP.	The size and the duration of air-carriage of respiratory droplets and droplet-nuclei	1946	Speaking, coughing, and sneezing	Descriptive, observational	Not reported	• Diameter of droplet-nuclei<br>• Size distribution of droplets by activity (speaking, coughing, sneezing)<br>• Duration of air-carriage of droplet-nuclei by their size	• Respiratory droplets varied in diameter from 1 to 2000 µms.<br>• No significant difference between the size distribution of droplet-nuclei produced by different types of activity<br>• More small nuclei created in more violent activities e.g. sneezing<br>• Larger nuclei the first to disappear from the air<br>• Droplets >8 µms disappeared from the air within 20 min, those >4 µms within 90 min<br>• Faster rate of disappearance when an electric fan was running<br>• Longest duration of air-carriage= 30 hours
Papineni RS, Rosenthal FS.	The size distribution of droplets in the exhaled breath of healthy human subjects	1997	Breathing, coughing, and talking	Descriptive, observational	n = 5.	• Size distribution of droplets produced by expired breath	1) Optical particle counter (OPC) measurement:<br>• Highest droplet concentration found for coughing and lowest for nose breathing<br>• 10-20% of total particle concentration were particles > 1µms in diameter<br>• Effects of mode (i.e. conditions: mouth breathing, nose breathing, coughing, and talking) not consistent across subjects<br>• Considerable inter-subject variability in particle size and concentration across modes<br>• Significant effects of mode and subject and significant mode-subject interaction shown through general linear model (mode and subject= independent variables; droplet concentration= dependent variables)

							• All 4 modes of breathing were significantly different from each other in the < 1 µms category<br>• In the > 1 µm category, coughing was significantly different from the other modes<br><br>2) Measurements with the analytical transmission electron microscope (AEM):<br>• 47 particles had original droplet size estimated to be 0.4 to 7.6 µms<br>• 30 droplets were estimated to have original size of > 1 µms
Zhu S, Kato S, Yang JH.	Study on transport characteristics of saliva droplets produced by coughing in a calm indoor environment	2006	Coughing	Descriptive study.	n = 3.	• Initial velocity of the coughed airflow<br>• Mass of coughed saliva<br>• Dispersion process of saliva droplets produced by coughing	• Initial velocity of coughed airflow 6 - 22m/s<br>• Mass of saliva ranged between 6-8mg/cough and averaged about 6.7mg/cough<br>• Saliva droplets contained in coughed airflow with diameters less than the diameter of the flour particles expected to disperse over a greater distance<br>• Saliva droplets ≤30µm expelled from the mouth soon dispersed throughout the room (no significant influence of gravity or inertia)<br>• Saliva droplets 50-200µm were most affected by gravity<br>• Saliva droplets > 300µm were affected more by inertia than by gravity and travelled further, especially saliva droplets of 500µm which could impact directly on the opposite person.
Yang S, Lee GW, Chen CM, Wu CC, Yu KP.	The size and concentration of droplets generated by coughing in human subjects	2007	Coughing	Experimental design	n = 54.	• Size distribution of droplet nuclei<br>• Size distribution and concentration of coughed droplets	• Total average size distribution of droplet nuclei 0.58-5.42 µm<br>• 82% of droplet nuclei centred in 0.74-2.12 µm range- these droplet nuclei likely contribute to airborne disease transmission<br>• Size distribution of droplets was 0.62-15.9µm

							• Pre-dominant size range of coughed droplets was large so coughed droplets may contribute to droplet transmission
Tang JW, Liebner TJ, Craven BA, Settles GS.	A schlieren optical study of the human cough with and without wearing masks for aerosol infection control	2009	Coughing	Descriptive study	n = 9.	• Speed of airflow released from coughs (measured via a velocity-magnitude contour and vector map)<br>• Shift distance of airflow	• Maximum average airspeed across early-staged cough was 8m/s<br>• Airflow patterns produced by unobstructed coughs (no mask): airflows fully turbulent; average total jet spreading angle 23.9 degrees<br>• Coughing without a mask produced a turbulent air jet extending across the schlieren field-of-view and well beyond it<br>• Standard surgical mask effectively blocked forward momentum of cough airflow jet and its aerosol content, but loose fit of mask margins allowed significant amount of air leakage through top, bottom and sides of mask<br>• N95 mask reduced leakage around the edges of the mask during coughing (tight seal) but increased pressure inside the mask- forcing more air through the front of the mask while decelerating it significantly
Nicas M, Jones RM.	Relative contributions of four exposure pathways to influenza infection risk	2009	Coughing	Modelling	Not reported.	• Overall infection risk<br>• Percentage of risk contributed by 4 exposure pathways as a function of virus concentration in saliva	• Contribution of each pathway varied depending on ratio between:<br>a) dose-response parameter for exposure to upper respiratory tract and<br>b) dose-response parameter for exposure to lower respiratory tract<br>• Ratio 3200/1: hand contact, respirable particle inhalation and droplet spray pathways contribute significantly to infection risk<br>• Ratio 1/1: hand contact pathway most important; inspirable particle inhalation and droplet spray significant but contributed less

Johnson GR, Morawska L.	The mechanism of breath aerosol formation	2009	Inhalation and exhalation.	Descriptive study.	n = 17.	• Aerosol size distribution<br>• Aerosol size diameter in range 0.5-20μm	• Deep breathing= significant concentration enhancement compared to normal breathing<br>• No significant difference in concentration enhancement between rapid and slow exhalation<br>• Deep exhalation prior to drawing a breath= increase in average concentration across all subjects by a factor of 5.5±3.5<br>• Deep exhalation produced increases in concentration compared to normal breathing; increase much greater with rapid inhalation than with rapid exhalation<br>• Distinctive erosion of particle concentration occurs, with move from large to smaller sizes as duration of breath-holding increases<br>- Suggests gravitational settling (larger particles that fall with greater terminal velocity disappear earlier)<br>- Evidence that aerosols formed primarily during inhalation rather than exhalation<br>• Positive significant correlation between concentration and subject age- breath aerosol production increases with age
Morawska LJ, Johnson GR, Ristovski ZD, Hargreaves M, Mengersen K, Corbett S, Chao CY, Li Y, Katoshevski D.	Size distribution and sites of origin of droplets expelled from the human respiratory tract during expiratory activities	2009	Cough, Breathing, Speech	Descriptive study.	n = 15.	• Modality and concentration of aerosol particles versus respiratory activities<br>• Modal diameter of droplet versus droplet age	• Large proportion of particles produced in modes with diameters <0.8μm at average concentrations up to 0.75/cm3<br>• Speech produced additional particles in modes near 3.5 and 5μm<br>- Modes became more prominent during sustained vocalization (average concentrations of 0.04 and 0.16/cm3, respectively)<br>• Average particle number concentrations produced during exhalation ranged from 0.1/cm3 for breathing to 1.1/cm3 for sustained vocalization<br>• Evaporation to equilibrium droplet size occurred within 0.8 seconds

Xie X, Li Y, Sun H, Liu L.	Exhaled droplets due to talking and coughing	2009	Talking, coughing	Descriptive study.	n = 7	• Droplet sizes and numbers<br>• Total mass of droplets	• Average number of droplets: Talking without food dye= 323; Talking w food dye= 315; Talking w food dye plus sugar solution= 273; Coughing= 108<br>• Droplet sizes for talking w food dye plus sugar: 15% <10 µm, 52% <50 µm, 80% <100 µm<br>• Droplet sizes when food dye was used: 5% <20 µm, 49% <50 µm, 83% <100 µm<br>• Droplet sizes when no food dye was used: 3% < 20 µm, 37% < 50 µm, 82% <100 µm<br>• Droplet sizes in coughing (no food dye): 2.5% < 20 µm, 1.4% <10 µm, 20% <50 µm, 64% <100 µm<br>• Coughing: 22.9mg of fluid obtained during 20 coughs (surgical facemask method), 85mg of fluid (plastic bag with tissue)<br>• Talking: 18.7mg (surgical mask), 79.4mg (plastic bag)
Stelzer-Braid S, Oliver BG, Blazey AJ, Argent E, Newsome TP, Rawlinson WD, Tovey ER.	Exhalation of respiratory viruses by breathing, coughing, and talking	2009	Breathing, talking, coughing.	Descriptive study.	n = 50 (33 symptomatic and 17 asymptomatic) including 10 children and 40 students.	• Detection of respiratory viruses in samples collected during breathing, talking, and coughing<br>• Presence of virus in exhaled samples.	• 12/25 subjects (virus-positive nasal mucus samples)= positive samples from breathing through mouth/ nose, 9= positive samples from talking, 2= positive samples from coughing<br>• 4 asymptomatic subjects= rhinovirus detected in nasal mucus, coughing sample, and breathing sample<br>• Subgroup of separate nasal and oral breathing samples collected: 4= virus in mucus and nose-breathing samples but not mouth-breathing samples; 1= influenza in mouth-breathing sample and rhinovirus in mucus sample<br>• Viable/live viruses detected in samples from breathing
Holmgren H, Ljungström E, Almstrand AC, Bake B, Olin AC.	Size distribution of exhaled particles in the range from 0.01 to 2.0 µm	2010	Breathing	Descriptive study.	n = 16.	• Particle concentrations during breathing<br>• Size distributions of particles during breathing	• Airway closure manoeuvre= significantly higher concentration of particles than tidal breathing<br>• Tidal breathing average size distribution of exhaled particles was log-normally distributed with a geometric mean of 0.07µm (SD = 2.0µm)

							• Breathing with airway closure- tidal particle mode still present, but additional stronger and broader maximum found between 0.2-0.5μm
Johnson GR, Morawska L, Ristovski ZD, Hargreaves M, Mengersen K, Chao CY, Wan MP, Li Y, Xie X, Katoshevski D, Corbett S.	Modality of human expired aerosol size distributions	2011	Speaking, coughing.	Descriptive study.	n = 15.	• Modality of the composite size distributions in different respiratory modes (B, L, and O)	• Three distinct droplet size distribution modes identified with count median diameters at 1.6, 2.5, and 145μm<br>• Voluntary cough modes located at 1.6, 1.7, 123μm<br>• Modes associated with 3 distinct processes: 1 occurring deep in the lower respiratory tract (B mode), 1 in larynx (L mode), and 1 in upper respiratory tract/oral cavity (O mode)
Hui DS, Chow BK, Chu L, Ng SS, Lee N, Gin T, Chan MT.	Exhaled Air Dispersion during Coughing with and without Wearing a Surgical or N95 Mask	2012	Coughing	Descriptive study	1 simulator.	• Expelled air dispersion distance along the median sagittal plane<br>• Lateral air dispersion	• Normal coughing mean exhaled air dispersion distance along median sagittal plane= 68cm, 30, and 15.1cm in non-mask, surgical mask, and N95 mask conditions<br>• Significant difference in air dispersion distance found across the conditions<br>• Significant leakage of air flow through mask-nasal bridge interface in upward direction, some downward leakage though lower edges with surgical mask<br>• N95 mask= less air leakage than surgical mask through the nasal bridge in upward direction<br>• Lateral dispersion distance of cough airflow= 27.9cm (surgical mask) and 15.0cm (N95)
Tang JW, Nicolle A, Pantelic J, Koh GC, De Wang L, Amin M, Klettner CA, Cheong DK, Sekhar C, Tham KW.	Airflow Dynamics of Coughing in Healthy Human Volunteers by Shadowgraph Imaging: An Aid to Aerosol	2012	Coughing	Descriptive study.	n = 20.	• Maximum detectable cough propagation distance (metres);<br>• Maximum derived velocity (m/s)<br>• Maximum detectable 2-D	• 10 females:<br>- Maximum detectable cough propagation distances= 0.16–0.55 m<br>- Maximum derived velocities= 2.2–5.0 m/s<br>- Maximum detectable 2-D projected areas= 0.010–0.11 m2<br>- Maximum derived expansion rates= 0.15–0.55 m2/s

	Infection Control					projected areas (m2)<br>• Maximum derived expansion rate (m2/s) (m2 = squared metre)	• 10 males:<br>- Maximum detectable cough propagation distances= 0.31–0.64 m<br>- Maximum derived velocities= 3.2–14 m/s<br>- Maximum detectable 2-D projected areas= 0.04–0.14 m2<br>- Maximum derived expansion rates= 0.25–1.4 m2/s<br>• Many cases show peak cough velocity shortly after onset of cough
Zayas G, Chiang MC, Wong E, MacDonald F, Lange CF, Senthilselvan A, King M.	Cough aerosol in healthy participants: fundamental knowledge to optimize droplet-spread infectious respiratory disease management	2012	Coughing	Descriptive study.	n = 45.	• Size and distribution of coughed droplets<br>• Impact of age, gender, weight, height, and body mass on aerosol droplets	• Cough generated droplets from 0.1-900 microns in size<br>• 99% of total droplets expelled when a healthy non-smoker coughs= droplets <10 µm, i.e. inhalable droplets<br>• Age, gender, weight, height, and body mass= no statistically significant effects on aerosol composition (size and number of droplets)
Lindsley WG, Pearce TA, Hudnall JB, Davis KA, Davis SM, Fisher MA, Khakoo R, Palmer JE, Clark KE, Celik I, Coffey CC.	Quantity and size distribution of cough-generated aerosol particles produced by influenza patients during and after illness	2012	Coughing	Descriptive.	n = 23. Of these, 9 were confirmed to have influenza on their 1st visit and returned for a second test after symptoms had resolved.	• Number of particles expelled per cough<br>• Volume of aerosol particles<br>• Volume of aerosol particles per litre of air coughed	• Number of particles expelled per cough:<br>- During influenza: Mean= 75400 particles/ cough, median = 46400, SD= 97300<br>- After recovered: Mean = 52200 particles/ cough, median = 8300, SD= 98600 (not significant)<br>• Volume of aerosol particles<br>- During influenza: 38.3 picoliters (pL)/cough<br>- After recovered: 26.4 pL/cough<br>• Volume of aerosol particles/L air coughed:<br>- During influenza: 14.9 pL/l<br>- After recovered: 8.5 pL/l

You SH, Chen SC, Wang CH, Liao CM.	Linking contact behavior and droplet patterns to dynamically model indoor respiratory infections among schoolchildren	2013	Coughing, talking	Cross-sectional study, and modelling study.	Survey study: n = 202; Droplet measurement experiment: n= 10.	• Contact behaviours of schoolchildren<br>• Size-dependent droplet number concentrations in coughing and talking<br>• Dynamics of transmission in schoolchildren population	• Estimated median number (SD) contacts/ person/ day= 9.44 ± 8.68 (grade 7), 10.12 ± 4.5 (grade 8), and 11.18 ± 7.98 (grade 9)<br>• Time-dependent droplet concentration highest at 32 minutes for both coughing (1.02 × 108 droplets/m3) and talking (8.52 × 107 droplets/m3)<br>• Small-diameter (0.3–0.4μm) droplets more numerous than large-diameter (>4 μm) droplets<br>• Total median droplet concentration= 9.01 × 107 (coughing) and 8.23 × 107 droplets/m3 (talking)<br>• Talking: size-dependent median number of droplets/ participant caused maximum of 8 infected persons at day 4 and 4 infected persons at day 3<br>• Coughing: size-dependent median number of droplets/ participant caused maximum of 10 infected persons at day 4 and 4 infected persons at day 4
Lindsley WG, Blachere FM, Beezhold DH, Thewlis RE, Noorbakhsh B, Othumpangat S, Goldsmith WT, McMillen CM, Andrew ME, Burrell CN, Noti JD.	Viable influenza A virus in airborne particles expelled during coughs versus exhalations	2016	Coughing, breathing	Descriptive study.	n = 61.	• Viable influenza virus in aerosol particles	• 28 (53%) subjects= aerosol particles containing viable influenza A virus produced during coughing<br>• 22 (42%)= aerosols with viable virus produced during exhalation<br>• 13= both cough aerosol and exhalation aerosol samples contained viable virus<br>• 15= positive cough aerosol but negative exhalation aerosol<br>• 9= positive exhalation aerosol but negative cough aerosol samples
Adhikari U, Chabrelie A, Weir M, Boehnke K, McKenzie E, Ikner L, Wang M, Wang Q, Young K, Haas CN, Rose J.	A Case Study Evaluating the Risk of Infection from Middle Eastern Respiratory Syndrome	2019	Coughing.	Modelling.	n = 1 modeling patient.	• Risk of MERS illness for the exposed population in hospital setting	• Nurses= highest daily risk of infection under a standard 6 air changes/hour for typical hospital room, followed by health care workers, family visitors<br>• Patients in same room= lowest daily risk of infection<br>• 90% of the uncertainty in risk characterization due to viral concentration in saliva

	Coronavirus (MERS-CoV) in a Hospital Setting Through Bioaerosols						• Risk intervention assessment showed respiratory masks= greater effect in reducing >90% the risks for all groups evaluated
Asadi S, Wexler AS, Cappa CD, Barreda S, Bouvier NM, Ristenpart WD.	Aerosol emission and superemission during human speech increase with voice loudness.	2019	Speaking, breathing	Descriptive study.	n = 48	• Relationship between particle dynamics and vocalization in different tasks<br>• Particle emission rate (N)<br>• Correlation between particle emission rate and vocal loudness/amplitude<br>• Size distribution of particles<br>• Correlation between size distribution of particles and vocal loudness/amplitude	• Particle release was highly correlated with vocalization: when vocalization started, number of particles increased rapidly then decreased to zero in nose breathing<br>• /a/ vocalization= 2 particles/cm3 of sampled air<br>• Particle emission rate increased from 6- 53 particles/second at the quietest and loudest vocal intensity, respectively<br>• Size distribution of particles not affected by vocal intensity/amplitude<br>• Variation across participants' particle emission rate- one participant= 200 particles/second at the higher amplitude; another= 1 particle/second at lower amplitudes<br>• No significant impact of temperature and humidity on particle emission rate or the mean particle diameter/size<br>• Some individual participants emitted many more particles than others<br>• Particle emission rate for speech significantly higher than all types of breathing (nose, mouth, deep-fast, and fast-deep breathing)
Lee J, Yoo D, Ryu S, Ham S, Lee K, Yeo M, Min K, Yoon C.	Quantity, size distribution, and characteristics of cough-generated	2018	Coughing	Descriptive study.	n = 12, of these 10 were confirmed as having a cold	• Particle number concentrations<br>• Size distribution	• Particles expelled/ cough while subjects had cold= 731,000 to 18,756,000 (mean: 4,914,600 particles/cough)<br>• Particles expelled/ cough after subjects recovered= 200,900 to 450,000

	aerosol produced by patients with an upper respiratory tract infection				(M = 5, F = 5).		• Surface area (SA) particles expelled/ cough when subjects had cold= 156,000 to 66,824,000 µm2 (mean: 7,210,000 µm2/cough)<br>• SA particles expelled/ cough after subjects recovered= 39,000 to 2,681,000 µm2 (mean: 521,000 µm2)<br>• Mean number particles/ cough and mean SA particles/ cough were higher in certain diameter ranges (< 100 nm, 100–300 nm, 420–1,000 nm, and 1.0–2.5 µm) when subjects had cold vs recovered<br>• 9/10 subjects= higher particle concentrations when ill
Asadi S, Wexler AS, Cappa CD, Barreda S, Bouvier NM, Ristenpart WD.	Effect of voicing and articulation manner on aerosol particle emission during human speech	2020	Speaking	Descriptive study.	n = 56	• Respiratory particle emission rate	• Certain phones had significantly higher particle production; /i/ produced more particles than /a/<br>• Disyllabic words including voiced plosive consonants had more particles than words with voiceless fricatives<br>• Particle emission rates positively correlated with vowel content of a phrase<br>• Particle emission decreased during phrases with a high proportion of voiceless fricatives
Georgiou GP, Kilani A.	The Use of Aspirated Consonants During Speech May Increase the Transmission of COVID-19	2020	Speaking languages with aspiration vs languages without aspiration	Theoretical assumption with examples.	Examples of Cases/million of population: 254.9 for languages with aspiration and 206 for languages without aspiration.	• Comparison of infected cases between languages with aspiration and languages without aspiration.	• No statistically significant differences in case numbers between languages with aspiration and languages without aspiration

Giovanni A, Radulesco T, Bouchet G, Mattei A, Révis J, Bogdanski E, Michel J.	Transmission of Droplet-Conveyed Infectious Agents Such As SARS-CoV-2 by Speech and Vocal Exercises During Speech Therapy: Preliminary Experiment Concerning Airflow Velocity	2020	Speaking	Descriptive study.	n =2.	• Exhaled airflow velocities	• Higher airflow velocities in loud and whispered voice<br>• Voiced consonant had higher velocities than vowels<br>• Voiceless consonants generated very fast airflows<br>• Semi-occluded vocal tract exercises generated airflows faster than loud speech<br>• Velocities decreased when voicing in the device

***Summary Table 2*:** Extracted Data from Review Articles

**Authors**	**Article title**	**Year**	**Review type**	**AGPs explored**	**Number of studies screened, excluded and reasons for exclusion**	**Methods used to assess risk of bias in individual studies**	**Strength of evidence assessment**	**Variables/Outcome measures under review**	**Main findings**
Tang JW, Li Y, Eames I, Chan PK, Ridgway GL.	Factors involved in the aerosol transmission of infection and control of ventilation in healthcare premises	2006	Review	Breathing, talking, coughing, sneezing, and singing.	Not reported.	Not mentioned.	No	• Droplets potentially leading to generation of infectious aerosol<br>• Survival of aerosolized pathogens in environment<br>• Transport/transmission characteristics of aerosols<br>• Role of inhaled pathogen dose and host's immune response in acquiring an infection<br>• Measures to control for airborne transmission of infectious diseases	• Droplets from talking, laughing, coughing, and sneezing potentially lead to generation of infectious aerosols<br>• Survival of aerosolized pathogens depends on environmental conditions- can vary with season and indoor environments<br>• Aerosols can be transmitted over short and long distances<br>• Agents which transmit infection over long distances usually transmit over short ranges and via direct contact<br>• Large droplets may become small droplets then droplet nuclei via evaporation<br>• Final inhaled pathogen dose and host's immune response determines whether infected<br>• Can restrict airborne transmission by controlling source of infection (e.g. quarantine), negative pressure ventilation systems, door designs, seal, use of PPE

Carlson AL, Budd AP, Perl TM.	Control of influenza in healthcare settings: early lessons from the 2009 pandemic	2010	Review	Talking	Not reported.	Not mentioned.	No.	• Transmission routes of influenza<br>• Intervention measures to prevent transmission	• 4 potential modes of influenza transmission= direct contact, indirect contact via fomites, droplet transmission, airborne transmission<br>• Droplet transmission= particles >5µm in diameter, fall to ground within 1m of source<br>• Airborne transmission caused by smaller particles- remain suspended in air, circulate<br>• Influenza infection can occur by experimental exposure to aerosolized particles but data on natural airborne transmission inconclusive<br>• Hand hygiene, wearing facemasks, gowns, and gloves to protect<br>• Applying physical measures reduced transmission of influenza<br>• Rates of influenza vaccination in HCP is low worldwide; rates of vaccination high when mandatory (99%)
Gralton J, Tovey E, McLaws ML, Rawlinson WD.	The role of particle size in aerosolised pathogen transmission: a review	2011	Review	Breathing, talking, sneezing, and coughing.	26 studies reviewed. Number of studies screened, excluded and reasons for exclusion	Not mentioned.	No.	• Droplet size/diameter<br>• Factors that influence particle size	• Infectious particles < 10µm more serious health implications (can penetrate lower respiratory tract to cause infection)<br>• Particles generated from respiratory activities 0.01-500µm, with particle size

					were not mentioned.				range of 0.05-500µm associated with infection.<br>• Few studies directly associate specific pathogen carriage with particular size range<br>• After expulsion, particle size influenced by host and extraneous factors
Zemouri C, de Soet H, Crielaard W, Laheij A.	A scoping review on bio-aerosols in healthcare and the dental environment	2017	Scoping review	Talking, breathing, sneezing, coughing.	5823 studies retrieved; duplicates and irrelevant records removed. 201 full text studies assessed for eligibility. 62 studies included. Reasons for exclusion provided.	Not mentioned.	No.	• Source of aerosols in dental clinics and hospital<br>• Microbial composition of bio-aerosols in hospital environment and dental clinics<br>• Hazard of a bio-aerosol	• Only one study reported solely on generation of bio-aerosols i.e. humans produce aeorosols by coughing and sneezing, level of evidence not provided<br>• Mainly referred to aerosol generations by hospital and dental procedures- level of evidence not provided
Mick P, Murphy R.	Aerosol-generating otolaryngology procedures and the need for enhanced PPE during the COVID-19 pandemic: a literature review	2020	Review	Literature of AGPs in ENT, no specific AGPs were the focus of the review.	Not reported.	Not mentioned.	No.	• Types of AGPs in ENT<br>• Evidence of enhanced PPE for AGPs in ENT	• Respiratory aerosols consist of droplet nuclei < 5µm diameter<br>• Droplets fall to ground at rates inversely proportional to size<br>• 10µm particles settle in 8.2min, 1.5h for 3µm particle, 12h for 1µm particle<br>• WHO considers cough to be aerosol-generating<br>• Average distribution of droplet sizes expelled during cough between 0.58-5.42µm

									• Aerosols are generated by pursed lip breathing and can be produced by normal breathing
Bolton L, Mills C, Wallace S, Brady MC, COVID LT, Group A.	Aerosol generating procedures, dysphagia assessment and COVID-19: A rapid review.	2020	Review	Coughing (Voluntary cough, reflexive cough.)	Not reported.	Not mentioned.	No.	• Route of transmission of COVID-19<br>• Role of aerosols in transmission of COVID-19<br>• Coughing and the risk to generate aerosols during cough<br>• Coughing during dysphagia assessment;<br>• AGPs<br>• Procedures that result in forceful coughing	• Emission of very small droplets from positive patients increases risk of airborne transmission<br>• Aerosols may remain in air for long time, travel over a distance, may cause infection if inhaled<br>• Definition of aerosols and droplets arbitrary- based on droplet size rather than measure of infection risk or transmission rate<br>• Respiratory droplet emissions from cough/ sneeze form complex droplet cluster across range of sizes, levels of respiratory system<br>• Distance travelled by coughed droplets depends on patient physiology, airflow, humidity, temp<br>• Saliva droplets emitted during forceful coughing important route for virus transmission<br>• Many COVID-19 patients w dysphagia predisposed to coughing in dysphagia assessments due to concomitant respiratory conditions
Viswanath A, Monga P.	Working through the COVID-19 outbreak: rapid	2020	Rapid review	Talking, coughing, sneezing.	Not reported.	Not mentioned.	No.	• Aerosol features<br>• Physical characteristics of coronavirus	• Aerosol spread from infected patients to HCWs via direct aerosol assault varies from

	review and recommendations for MSK and allied heath personnel							• Filtering characteristics of some mask types<br>• Direct exposure via contact	low level contamination (e.g. talking) to high level (e.g. coughing, sneezing)<br>• SARS-CoV2 detectable in aerosols for up to 3h- hence aerated rooms safer than closed spaces<br>• Size of coronavirus about 100nm (0.1μm); particles pass through most mask filters e.g. N95<br>• Particles < 300nm do not travel in a linear direction: zig-zag movement so often trapped in filters with bigger mesh sizes<br>• N95 masks recommended for medium risk clinical encounters<br>• Recommend positive pressure suits for high risk<br>• Virus can stay alive on respirator surface (3 days)
Wilson NM, Norton A, Young FP, Collins DW.	Airborne transmission of severe acute respiratory syndrome coronavirus-2 to healthcare workers: a narrative review	2020	Narrative review	Coughing, sneezing	Not reported.	Not mentioned.	No.	• Not clearly defined.	• Airborne viral particles form through open-close cycling of glottic structures (>1μm diameter), shearing forces (high velocity gas flow) (2-5μm), open-close cycling of terminal bronchiole airways (< 1μm)<br>• Exhaled particles come from lower respiratory tract<br>• Viral growth in-medium can occur from particles < 5μm<br>• Infected human subjects produce more particles when coughing compared to healthy

									• Infected particles < 5-20µm can reach respiratory portion of airway<br>• Gas flow velocities vary with type of exhalation<br>• Explosive shearing forces (coughing, sneezing) produce highest number particles and variable sized particles, but significant numbers and size range produced in talking, tidal volume breathing<br>• Exhaling to closing capacity strongly correlated with significant aerosol production<br>• Sneeze and cough can form a turbulent multiphases gas cloud which extend lifespan of droplet, cloud can travel up to 8m, droplets then evaporate to form droplet nuclei –suspended in air for hours<br>• Larger particles travel shorter distances, smaller particles remain suspended indefinitely<br>• Site of particle deposition in the airway depends on variety of factors, subject-specific variables, disease, etc.<br>• 500-ml breath draws gas approx. 10cm from mouth<br>• 5-µm size threshold of WHO is an over-simplification of multifactorial mechanisms governing aerosol dispersal and deposition<br>• Frequent cough and dyspnoeic spontaneous respiration are

									natural aerosol generators associated with transmission of SARS-CoV-1
Xu R, Cui B, Duan X, Zhang P, Zhou X, Yuan Q.	Saliva: potential diagnostic value and transmission of 2019-nCoV	2020	Review	Talking, coughing, sneezing.	Not reported.	Not mentioned.	No.	• Characteristics of saliva as droplets and aerosols<br>• Diagnostic values of saliva in SARS-CoV2<br>• Transmission of SARS-CoV2 via saliva	• Droplet travel distance largely determined by size<br>• Most communicable respiratory infections transmitted via large droplets in short distance or contact with contaminated surfaces<br>• Small droplets likely to evaporate into droplet nuclei (diameter < 10μm) in favourable environments, then have potential for long-distance aerosol transmission<br>• Saliva droplets generated when breathing, talking, coughing, or sneezing<br>• Amount, distance, size of saliva droplets varies among people<br>• 3000 saliva droplet nuclei in one cough- nearly equals amount produced in 5-mins of talking<br>• One normal exhalation can generate saliva droplets reaching 1m in air<br>• Aerosols are suspension of particles in air, liquid, or solid; size from 0.001 to above 100μm
Pasnick S, Carlos WG, Dela Cruz CS, Gross	SARS-CoV-2 Transmission and the Risk of Aerosol	2020	Fact sheet	Sneezing, coughing, speaking, singing, laughing.	Not reported.	Not mentioned.	No.	• Routes of transmission of SARS-CoV2<br>• Risks of SARS-CoV2 transmission in aerosol generating procedures	• SARS-CoV2 can remain infectious on surfaces up to 72h, in aerosols up to 3h<br>• AGPs have potential to generate aerosols and droplets

JE, Garrison G, Jamil S.	Generating Procedures								that can spread respiratory pathogens<br>• Much information regarding true aerosolizing potential of various procedures and role in transmitting respiratory diseases- speculative<br>• Most data regarding risks to healthcare workers extrapolated from epidemiological data collected in infectious outbreaks<br>• Studies limited by sample size and study design
Naunheim MR, Bock J, Doucette PA, Hoch M, Howell I, Johns MM, Johnson AM, Krishna P, Meyer D, Milstein CF, Nix J.	Safer Singing During the SARS-CoV-2 Pandemic: What We Know and What We Don't	2020	Review	Singing	Not reported.	Not mentioned.	No.	• Transmission pathways of SARS-CoV2<br>• Respiratory and phonatory activities that generate droplets and aerosols<br>• Environmental factors that affect the risk of transmission<br>• Role of personal protective equipment in prevention of virus spread	• Rapid spread of COVID-19 around the world suggests aerosols might play a more important role than initially suspected<br>• Certain individuals are "speech super-emitters" who generate significantly more aerosols particles than others<br>• Louder phonation results in greater aerosol generation<br>• Airflow is a key element in all singing styles- one study has shown that whispered and breathy phonation produce significantly greater airflow than normal phonation at all loudness levels<br>• Singing with more resonant voice and less airflow could be less likely to transmit disease

									• Unknown how aerosol production varies according to voice type, vocal register, or vocal style<br>• Unlikely to completely eliminate aerosol burden<br>• Unknown whether greater use of vital capacity in singing has impact on creation of aerosols as compared to singing techniques that use shallower breaths and a shorter vocal tract
Qureshi Z, Jones N, Temple R, Larwood JP, Greenhalgh T, Bourouiba L.	What is the evidence to support the 2-metre social distancing rule to reduce COVID-19 transmission?	2020	Rapid review	Exhalation, speech, coughing, sneezing	Not reported.	Not mentioned.	No.	• Evidence for the distance that respiratory droplets of different sizes can travel<br>• The factors that affect the distance that respiratory droplets can travel/spread	• Droplets can travel >2m depending on factors such as droplet size, type of respiratory/phonatory activities, and indoor environment<br>• Current level of evidence is low

Summary Table 3: Extracted Data from Clinical Guidelines

**Organisation responsible for development**	**Title of document**	**Year**	**Focus of guidelines**	**Aims of guideline and specific objectives**	**AGPs examined**	**Target population and subgroups**	**Summary of recommendations provided in guideline**	**Is this guideline based on new or existing systematic reviews?**
French Society of Otorhinolaryngology, Head and Neck Surgery (SFORL)	Guidelines of clinical practice for the management of swallowing disorders and recent dysphonia in the context of the COVID-19 pandemic	2020	Management of swallowing disorders and recent dysphonia.	To provide recommendations regarding the management of swallowing disorders and the assessment of acute dysphonia.	Coughing.	HCWs working in ENT and SLP, patients with swallowing disorders and/or recent/acute dysphonia, and caregivers.	• Vocal rehabilitation should not be considered urgent in the current epidemic context<br>• Tele-rehabilitation should be followed through wherever possible<br>• Same precautions should apply to all patients<br>• Only swallowing disorders management or exploration of recent dysphonia regarded as impossible to post-pone should be maintained- should be validated by team discussions or rely on official recommendations from health authorities<br>• Procedures considered as very high risk of contamination: clinical and flexible endoscopic swallowing assessments, flexible endoscopies, insertions of nasogastric tubes and video fluoroscopic swallowing exams<br>• When risky procedures cannot be postponed, following PPE is recommended: protective glasses, FFP2 (N95) mask, cap, gloves and gown	No.
Speech Pathology Australia	UPDATED Speech Pathology Australia guidance for	2020	Service delivery, clinical procedures,	The guideline aimed to provide in-principle guidance relating to speech pathology	Coughing, talking, loud voicing, singing,	Speech pathologists and their patients.	• FEES assessments with COVID-19 suspected or positive clients not recommended	No.

	service delivery, clinical procedures and infection control during COVID-19 pandemic		and infection control.	service delivery, clinical procedures and infection control to minimise transmission.	voice assessment tasks.		• Recommended that speech pathologists consider risk of droplet/aerosol exposure prior to undertaking any clinical service<br>• Speech Pathology Australia recommends airborne precautions are used for all clients when there is potential exposure to aerosols regardless of known COVID-19 status of the client<br>• Surgical to be used AT ALL TIMES (within 1.5m of patient), but need to recognise when additional / higher levels of transmission precautions and PPE is needed<br>• Clear face shield NOT considered to provide same protection as a face mask<br>• SLPs to determine if a clinical service is appropriate to provide in-person when wearing a mask i.e. consider telepractice	
Royal College of Speech Language Therapists	RCSLT guidance on reducing risk of transmission, use of personal protective equipment (PPE) in the context of COVID-19.	2020	Prevention of the transmission of COVID-19 and the use of personal protective equipment (PPE).	To support all RCSLT members, regardless of where they work and who they are employed by (public sector, charity sector, or independent sector) to make informed decisions about safe ways of working during the COVID-19 pandemic	Coughing, sneezing, singing, forceful blowing, Lee Silverman Voice Therapy.	Speech pathologists.	• Use new ways of working as telehealth, coaching/training for SLPs and others, cohorting/bubbles<br>• Change working practice in both inpatient and outpatient settings<br>• Use of personal protective equipment in reducing the risk of transmission of COVID-19	Yes. Limited literature provided.
