# Supplementary material for: Aerosol-generating behaviours in speech pathology clinical practice: A systematic literature review
Source: PLoS One. 2021 Apr 28;16(4):e0250308. doi: 10.1371/journal.pone.0250308 (PMC8081183; doi:10.1371/journal.pone.0250308)
Supplement: S4 File — (PDF) [file pone.0250308.s005.pdf]

Systematic review

To edit the record click *Start an update* below. This will create a new version of the record - the existing version will remain unchanged.

1. * Review title.

Give the title of the review in English

Aerosol-generating behaviours in voice and speech: a systematic review of the literature

2. Original language title.

For reviews in languages other than English, give the title in the original language. This will be displayed with the English language title.

3. * Anticipated or actual start date.

Give the date the systematic review started or is expected to start.

07/05/2020

4. * Anticipated completion date.

Give the date by which the review is expected to be completed.

30/11/2020

5. * Stage of review at time of this submission.

Tick the boxes to show which review tasks have been started and which have been completed.
Update this field each time any amendments are made to a published record.

Reviews that have started data extraction (at the time of initial submission) are not eligible for inclusion in PROSPERO.
If there is later evidence that incorrect status and/or completion date has been supplied, the published PROSPERO record will be marked as retracted.

This field uses answers to initial screening questions. It cannot be edited until after registration.

The review has not yet started: No

Review stage	Started	Completed
Preliminary searches	Yes	Yes
Piloting of the study selection process	Yes	Yes
Formal screening of search results against eligibility criteria	Yes	Yes
Data extraction	Yes	Yes
Risk of bias (quality) assessment	Yes	Yes
Data analysis	Yes	Yes

Provide any other relevant information about the stage of the review here.

Review has been finalised and submitted for publication.

Review has been finalised and submitted for publication.

6. * Named contact.

The named contact is the guarantor for the accuracy of the information in the register record. This may be any member of the review team.

A/Prof Catherine Madill

Email salutation (e.g. "Dr Smith" or "Joanne") for correspondence:
Cate

7. * Named contact email.

Give the electronic email address of the named contact.

cate.madill@sydney.edu.au

8. Named contact address

PLEASE NOTE this information will be published in the PROSPERO record so please do not enter private information, i.e. personal home address

Give the full institutional/organisational postal address for the named contact.

9. Named contact phone number.

Give the telephone number for the named contact, including international dialling code.

10. * Organisational affiliation of the review.

Full title of the organisational affiliations for this review and website address if available. This field may be completed as 'None' if the review is not affiliated to any organisation.

The University of Sydney

Organisation web address:
https://www.sydney.edu.au/

11. * Review team members and their organisational affiliations.

Give the personal details and the organisational affiliations of each member of the review team. Affiliation refers to groups or organisations to which review team members belong.
NOTE: email and country now MUST be entered for each person, unless you are amending a published record.

Miss Antonia Chacon. The University of Sydney
Dr Duong Nguyen. The University of Sydney
Professor Patricia McCabe. The University of Sydney
Assistant/Associate Professor Catherine Madill. The University of Sydney

12. * Funding sources/sponsors.

Details of the individuals, organizations, groups, companies or other legal entities who have funded or sponsored the review.

None.

Grant number(s)
State the funder, grant or award number and the date of award

13. * Conflicts of interest.

List actual or perceived conflicts of interest (financial or academic).

None

14. Collaborators.

Give the name and affiliation of any individuals or organisations who are working on the review but who are not listed as review team members. **NOTE: email and country must be completed for each person, unless you are amending a published record.**

15. * Review question.

State the review question(s) clearly and precisely. It may be appropriate to break very broad questions down into a series of related more specific questions. Questions may be framed or refined using PI(E)COS or similar where relevant.

Which voice, resonance and motor speech tasks performed in speech pathology practice are considered to be aerosol-generating?

16. * Searches.

State the sources that will be searched (e.g. Medline). Give the search dates, and any restrictions (e.g. language or publication date). Do NOT enter the full search strategy (it may be provided as a link or attachment below.)

Databases searched: OVID MEDLINE, OVID Embase, Scopus, Web of Science, CINAHL and PubMed Central

Grey literature searched: ProQuest (unpublished dissertations), The Centre for Evidence-Based Medicine, COVID-Evidence and Speech Pathology national bodies inclusive of Speech Pathology Australia (SPA), The Royal College of Speech and Language Therapists (RCSLT) and The American Speech Language Hearing Association (ASHA).

Search restrictions: English language only, human studies only, restricted to dates 1940-present.

The search strategy is detailed in the protocol (see attached PDF document, Field #34 (Reference and/or URL for published protocol)).

17. URL to search strategy.

Upload a file with your search strategy, or an example of a search strategy for a specific database, (including the keywords) in pdf or word format. In doing so you are consenting to the file being made publicly accessible.

Or provide a URL or link to the strategy. Do NOT provide links to your search **results**.

https://www.crd.york.ac.uk/PROSPEROFILES/186902_STRATEGY_20201203.pdf

Yes I give permission for this file to be made publicly available

18. * Condition or domain being studied.

Give a short description of the disease, condition or healthcare domain being studied in your systematic review.

Speech pathology clinicians and patients.
Risk of disease transmission through aerosol generation.

19. * Participants/population.

Specify the participants or populations being studied in the review. The preferred format includes details of both inclusion and exclusion criteria.

Inclusion: the general adolescent and adult human population aged 12 years and older.

Exclusion: populations with specific diseases, conditions, or disorders that do not involve a component of voice, resonance or motor speech impairment.

Exclusively inpatient hospital environments that do not reflect the typical proceedings of voice, resonance and/or motor speech assessments and/or interventions that occur in clinic office settings will also be excluded.

20. * Intervention(s), exposure(s).

Give full and clear descriptions or definitions of the interventions or the exposures to be reviewed. The preferred format includes details of both inclusion and exclusion criteria.

The only speech pathology tasks/interventions to be analysed in this review will be those completed in voice, resonance and motor speech assessments and interventions.

The specific tasks involved in each of these areas have been listed as search terms in the attached protocol (see PDF document, Field #34 (Reference and/or URL for published protocol)).

Exclusively inpatient hospital environments that do not reflect the typical proceedings of voice, resonance and/or motor speech assessments and/or interventions that occur in clinic office settings will be excluded.

21. * Comparator(s)/control.

Where relevant, give details of the alternatives against which the intervention/exposure will be compared (e.g. another intervention or a non-exposed control group). The preferred format includes details of both inclusion and exclusion criteria.

Not applicable.

22. * Types of study to be included.

Give details of the study designs (e.g. RCT) that are eligible for inclusion in the review. The preferred format includes both inclusion and exclusion criteria. If there are no restrictions on the types of study, this should be stated.

All study designs bar those that don't have any cited literature evidence, opinion/ commentaries and any papers not published/ accessible in the English language.

23. Context.

Give summary details of the setting or other relevant characteristics, which help define the inclusion or exclusion criteria.

There will be no exclusions based on types of settings, unless the settings described relate exclusively to inpatient hospital environments that do not reflect the typical proceedings of voice, resonance and/or motor speech assessments and/or interventions that occur in clinic office settings.

24. * Main outcome(s).

Give the pre-specified main (most important) outcomes of the review, including details of how the outcome is defined and measured and when these measurement are made, if these are part of the review inclusion criteria.

To investigate the aerosol-generating properties of different voice, resonance and motor-speech clinical tasks in a speech pathology setting.

* Measures of effect
Nil meta-analysis nor quantitative measures of effect used owing to diversity of collected data, outcomes, etc. The Synthesis Without Meta-analysis (SWiM) guidelines were used to inform the overall synthesis and summary of findings across all included articles.

25. * Additional outcome(s).

List the pre-specified additional outcomes of the review, with a similar level of detail to that required for main outcomes. Where there are no additional outcomes please state 'None' or 'Not applicable' as appropriate to the review

Aerosol characteristics related to risk of infectious disease transmission involved in voice, resonance and motor speech tasks in Speech Pathology assessment and treatment.

* Measures of effect
Nil meta-analysis nor quantitative measures of effect used owing to diversity of collected data, outcomes, etc. The Synthesis Without Meta-analysis (SWiM) guidelines were used to inform the overall synthesis and summary of findings across all included articles.

26. * Data extraction (selection and coding).

Describe how studies will be selected for inclusion. State what data will be extracted or obtained. State how this will be done and recorded.

Titles and abstracts of the studies retrieved during the searches were screened by two independent reviewers for eligibility against the inclusion criteria.
Studies and reports that potentially met the criteria were retrieved in full, and their citation details imported into the Covidence systematic review software.
The full texts of the selected citations were then assessed in detail against the inclusion criteria by two independent reviewers.
Titles and abstracts that did not meet the study criteria were removed, with the reasons for exclusions at the full text screening stage being recorded and reported in the review.
Any records which discussed the same study were grouped together.
Any disagreements were resolved through discussion, and/or through the involvement of a third reviewer.
The results of the search will be reported in full in the final systematic review, and presented in a PRISMA flow diagram.

Data was extracted by two independent reviewers from the studies selected for inclusion. Extracted data included specific details about the study population, concepts, contexts, methods and key findings relevant to the review question, amongst other domains.
The draft data extraction tool was trialed on a small number of studies to ensure that all relevant information was extracted, and was then modified as necessary (with details regarding the modifications detailed in the systematic review).
Any disagreements between the reviewers were resolved through discussion, or the involvement of a third reviewer.

27. * Risk of bias (quality) assessment.

State which characteristics of the studies will be assessed and/or any formal risk of bias/quality assessment tools that will be used.

The original study protocol planned to use the Cochrane Risk of Bias assessment to assess the risk of bias and quality of each of the collated records.

Through discussion with members of the research team it was decided that this tool did not enable an equitable means of assessing the quality of the range of papers collated, which includes research studies, review articles and clinical guideline documents. It was decided that the most valid means of assessing each paper's quality and potential risk of bias was to use the GRADE assessment tool and associated GRADEpro app. This site enabled each study to be evaluated against a consistent range of outcomes to determine the certainty of the record's evidence, and included a component enabling risk of bias assessment.

28. * Strategy for data synthesis.

Describe the methods you plan to use to synthesise data. This **must not be generic text** but should be **specific to your review** and describe how the proposed approach will be applied to your data.
If meta-analysis is planned, describe the models to be used, methods to explore statistical heterogeneity, and software package to be used.

The collected studies demonstrate high levels of heterogeneity, rendering a meta-analysis impossible. The Synthesis Without Meta-Analysis (SWiM) reporting guidelines facilitated our team's synthesis of the collected data and promoted clear reporting of the result findings.

Our summary will be an integrative analysis, involving both narrative and statistical techniques to reflect the anticipated diversity of evidence to be collected from this review. This will involve use of a summary table similar to that detailed in the protocol document to report our findings, followed by categorisation of data collected in similar concept areas (e.g. according to the range of aerosol-generating procedures), and the thematic analysis of the findings.

29. * Analysis of subgroups or subsets.

State any planned investigation of ‘subgroups’. Be clear and specific about which type of study or participant will be included in each group or covariate investigated. State the planned analytic approach.

No particular participant subgroups were found from our literature search of AGPs in SLP clinical procedures. However, our presentation of the result findings will be organised based on categorisation of common themes, such as all literature examining coughing as an AGP being grouped together with data synthesised.

30. * Type and method of review.

Select the type of review, review method and health area from the lists below.

Type of review

Cost effectiveness	No

Diagnostic	No
Epidemiologic	Yes
Individual patient data (IPD) meta-analysis	No
Intervention	No
Meta-analysis	No
Methodology	No
Narrative synthesis	Yes
Network meta-analysis	No
Pre-clinical	No
Prevention	Yes
Prognostic	No
Prospective meta-analysis (PMA)	No
Review of reviews	No
Service delivery	Yes
Synthesis of qualitative studies	No
Systematic review	Yes
Other	No

Health area of the review

Alcohol/substance misuse/abuse	No
Blood and immune system	No
Cancer	No
Cardiovascular	No
Care of the elderly	No
Child health	No
Complementary therapies	No
COVID-19	Yes

For COVID-19 registrations please tick all categories that apply. Doing so will enable your record to appear in area-specific searches

Chinese medicine

Diagnosis

Epidemiological

Genetics

Health impacts

Mental health

PPE

Prognosis

Public health

Rehabilitation

Service delivery

Transmission

Treatments

Vaccines

Other

Crime and justice	No
Dental	No
Digestive system	No
Ear, nose and throat	No
Education	No
Endocrine and metabolic disorders	No
Eye disorders	No
General interest	No
Genetics	No
Health inequalities/health equity	No
Infections and infestations	Yes
International development	No
Mental health and behavioural conditions	No

Musculoskeletal	No
Neurological	No
Nursing	No
Obstetrics and gynaecology	No
Oral health	No
Palliative care	No
Perioperative care	No
Physiotherapy	No
Pregnancy and childbirth	No
Public health (including social determinants of health)	No
Rehabilitation	No
Respiratory disorders	Yes
Service delivery	Yes
Skin disorders	No
Social care	No
Surgery	No
Tropical Medicine	No
Urological	No
Wounds, injuries and accidents	No
Violence and abuse	No

31. Language.

Select each language individually to add it to the list below, use the bin icon to remove any added in error.

There is an English language summary.

32. * Country.

Select the country in which the review is being carried out. For multi-national collaborations select all the countries involved.

Australia

33. Other registration details.

Name any other organisation where the systematic review title or protocol is registered (e.g. Campbell, or The Joanna Briggs Institute) together with any unique identification number assigned by them.
If extracted data will be stored and made available through a repository such as the Systematic Review Data Repository (SRDR), details and a link should be included here. If none, leave blank.

34. Reference and/or URL for published protocol.

If the protocol for this review is published provide details (authors, title and journal details, preferably in Vancouver format)

https://www.crd.york.ac.uk/PROSPEROFILES/186902_PROTOCOL_20200518.pdf

No I do not make this file publicly available until the review is complete

35. Dissemination plans.

Do you intend to publish the review on completion?

Yes

We plan to publish this review in PLOS One.
We will also be emailing contacts from The Royal College of Speech and Language Therapists, American Speech Language Hearing Association and Speech Pathology Australia amongst others, who have indicated an interest in the outcomes of the review.

36. Keywords.

Give words or phrases that best describe the review. Separate keywords with a semicolon or new line. Keywords help PROSPERO users find your review (keywords do not appear in the public record but are included in searches). Be as specific and precise as possible. Avoid acronyms and abbreviations unless these are in wide use.

Aerosol; COVID-19; Voice; Speech; Cough; Systematic Review

37. Details of any existing review of the same topic by the same authors.

If you are registering an update of an existing review give details of the earlier versions and include a full bibliographic reference, if available.

38. * Current review status.

Update review status when the review is completed and when it is published.
New registrations must be ongoing so this field is not editable for initial submission.

Review_Completed_not_published

39. Any additional information.

Provide any other information relevant to the registration of this review.

Currently awaiting PLOS One submission review.

40. Details of final report/publication(s) or preprints if available.

Leave empty until publication details are available OR you have a link to a preprint (NOTE: this field is not editable for initial submission).
List authors, title and journal details preferably in Vancouver format.
